# Oral Presentations 1

**DOI:** 10.1038/sj.bjc.6601926

**Published:** 2004-06-22


British Journal of Cancer (2004) 91(Suppl 1), S8 – S23
© 2004 Cancer Research UK All rights reserved 0007 – 0920/04 $30.00
www.bjcancer.com

### ORALS PRESENTATIONS 1

### 1.1

### PHASE I/II STUDY OF FRACTIONATED RADIOIMMUNOTHERAPY (RIT) IN RELAPSED LOW GRADE NON-HODGKIN'S LYMPHOMA (NHL).

M. Bayne , M. Zivanovic , Y. Du , A. Tutt , G. Mead , P. Johnson , V. Lewington , T. Illidge
CR UK Wessex Medical Oncology Unit, University of Southampton., Southampton, United Kingdom

RIT in "low grade" NHL produces high objective response rates with durable complete responses. The most impressive results, suggesting that there may be a radiation dose response for RIT, have been reported in clinical studies using high myeloablative dose RIT followed by peripheral blood stem cell transplant (PBSCT). The development of a human anti-mouse antibody response usually prevents more than one administration of RIT using the commercially available murine radioimmunoconjugates $^{131}$I tositumomab (Bexxar$^{TM}$) and $^{90}$Y ibritumomab tiuxetan (Zevalin$^{TM}$). Using a radioimmunoconjugate derived from the chimeric mAb rituximab, multiple or fractionated treatments are possible. Fractionation may enable higher cumulative doses of RIT to be delivered without the need for PBSCT. Using a dose escalation protocol this study tests the safety and efficacy of fractionated RIT in relapsed CD20 positive NHL using 2 fractions of $^{131}$I labelled rituximab preceded by an induction course of unlabelled rituximab. All 8 patients in the first 2 dose cohorts have responded with acceptable toxicity and results from 12 patients will be available at the time of presentation.
Sequential pharmacokinetic analyses have identified wide variation in the effective half-life of $^{131}$I-rituximab not only between patients but also within the same patient over the course of the treatment protocol. The mean effective half-life of $^{131}$I rituximab increases from 43 hours prior to induction rituximab to 106 hours prior to the second fraction of $^{131}$I-rituximab. An anti-rituximab idiotype monoclonal antibody (mAb) has been generated to enable serial analysis of serum rituximab concentrations. This tool has aided analysis of the impact of induction unlabelled rituximab, the pre-dose and fractionation on the clearance and biodistribution of the radioimmunoconjugate. Our results indicate a need to individualise the scheduling of RIT in order to improve the biodistribution of radioimmunoconjugate and maximize the therapeutic ratio.

### 1.2

### A PHASE I/II TRIAL OF ANTIBODY DIRECTED ENZYME PRODRUG THERAPY (ADEPT) WITH MFECP1 AND ZD2767P

A Mayer , R Francis , SK Sharma , C Sully , S Parker , B Tolner , N Griffin , M Germain , P Beckett , B Tolner , G Boxer , A Green , KA Chester , RHJ Begent
Department of Oncology, Royal Free Campus, University College London, London, United Kingdom

Antibody-directed enzyme prodrug therapy (ADEPT) is based on administration of an anti-tumour antibody enzyme complex followed by prodrug, which is converted to active drug at the tumour site. We report interim results of the Phase I study of single administration of ADEPT with MFECP1, a fusion protein of carboxypeptidase G2 (CPG2) and the anti-CEA scFv antibody MFE-23, and the bis-iodo phenol mustard prodrug ZD2767P. MFECP1 was developed to fulfil the design criteria of tumour localisation with rapid clearance.
Twenty-eight patients (15 male, 13 female; median age 64 years; range 35-78 years) with unresectable, locally recurrent or metastatic histologically proven colorectal or other CEA expressing cancer (serum CEA <1000ug/l) were included, 27 have completed the study. Patients #1-23 received 5000U/m$^2$ MFECP1 and patients #24-28 3000U/m$^2$ MFECP1, ZD2767P was escalated in two-fold increments from 12.42mg/m$^2$ x3 to 1075mg/m$^2$ x3.
MFECP1 cleared rapidly via the liver, the alpha half life was 0.5h and 0.44h and the beta half life 4.6h and 1.96h for 5000U/m$^2$ MFECP1 and 3000U/m$^2$ MFECP1, respectively. Based on SPECT imaging CPG2 in tumour was estimated to be 0.18U/g, 0.07U/g and 0.02U/g after 4h, 20h and 40h, respectively. Localisation was confirmed by immunohistochemistry up to 17 h after administration of MFECP1, prodrug activation by comet assay. Dose limiting toxicity was seen for the combination of 5000U/m$^2$ MFECP1 and 1075mg/m$^2$ x3 and 3000U/m$^2$ MFECP1 and 537.5mg/m$^2$. The combination of 3000U/m$^2$ MFECP1 and 268.8mg/m$^2$ was found to be safe and well tolerated. 0/27 patients had detectable HAMA, 10/27 detectable HACA. 9/27 patients achieved SD with 1 patient showing a 10% reduction of tumour diameter, 16 PD and 2 were not evaluable.
In conclusion, DLT and MTD for the combination of MFECP1 and ZD2767P were determined in the current phase I trial, efficacy will be tested after giving repeated treatment. MFECP1 cleared rapidly, localised in tumour and has less immunogenic potential than previous antibody enzyme conjugates.

### 1.3

### PHASE II RANDOMISED DISCONTINUATION STUDY OF BAY 43-9006 IN PATIENTS WITH ADVANCED MELANOMA

T Ahmad [1], R Marais [2], L Pyle [1], M James [1], B Schwartz [3], M Gore [1], T Eisen [1]
[1] Royal Marsden Hospital, London, United Kingdom, [2] Institute of Cancer Research, London, United Kingdom, [3] Bayer Pharmaceutical Corporation, West Haven, United States

**Background** Approximately 70% of melanomas have an activating mutation in BRAF, leading to elevated kinase activity and cellular proliferation. BAY 43-9006 is a potent RAF kinase inhibitor that retards the growth of human melanoma xenografts. This phase II study aims to determine the clinical benefit and toxicity of BAY 43-9006 in advanced melanoma and to correlate these findings with BRAF status of the patients and their tumours.
**Methods** Patients with stage IV refractory melanoma were treated with BAY 43-9006 400mg BD orally for 12 weeks after baseline imaging and biopsy of tumour and normal skin. At the 12 week reassessment, patients with objective tumour responses continued BAY 43-9006 until disease progression or unacceptable toxicity. Patients with stable disease were randomised in a double-blind manner between BAY 43-9006 and placebo and followed for on-going response. In the event of disease progression in this cohort, the code was broken and those receiving placebo were rechallenged with BAY 43-9006. Tumour responses were evaluated by CT/MRI at 12 weeks and every 6 weeks thereafter and tumour biopsies taken at 0, 4, 12 weeks for sequencing BRAF and identification of downstream activity of RAF kinase.
**Results** Twenty patients were enrolled between October 2002 and April 2003, the median age was 52 years.At week 12, 1 partial response and 3 stable disease were seen. Fifteen patients had progressive disease before or at 12 weeks. Grade 3 skin toxicity was observed in 5 patients and 2 patients developed hypertension requiring intervention.There was no haematological toxicity and all toxicity was reversible. Laboratory analysis of tumours to determine BRAF status will be presented.
**Conclusion** BAY 43-9006 has very modest activity as a single agent. Preliminary data from combination studies are promising and studies are ongoing.The drug is well-tolerated and the determination of BRAF status in patients may allow correlation with clinical behaviour of tumours and efficacy of BAY 43-9006.

### 1.4

### INITIAL PHASE 1 STUDY OF XR5944, A NOVEL DNA AND RNA TARGETING AGENT

EM Rankin , D Bissett , M Nicolson , H Thomas , J Steiner , M Cooper , G Mellows , G Allen , D Norris , J Waterfall
[1] United Kingdom, Division of Cancer Medicine, University of Dundee, Dundee, [2] United Kingdom, Grampian Universities NHS Trust, Aberdeen, [3] United Kingdom, St Lukes Cancer Centre, Guildford, [4] United Kingdom, Xenova Ltd, Slough, [5] United Kingdom, Millennium Pharmaceuticals, Cambridge

**Background:** XR5944 (MLN944) is a novel DNA/RNA targeting agent with a mechanism of action distinct from that of currently available cytotoxic chemotherapeutic agents. XR5944 binds to the major groove of DNA and inhibits RNA synthesis with an $EC_{50}$ = 3 nM. Because of XR5944's unique mechanism of action and activity across several human tumor cell lines and xenograft models, we have initiated a phase I clinical trial of this compound in patients with advanced, treatment-refractory solid tumors. XR5944 is given as a single 30 minute intravenous infusion repeated once every 21-28 days. The starting dose was 3.6 mg/m$^2$, which is a NOAEL (no observable adverse effect level) in the most sensitive species and is just below one-tenth of the MTD. Preclinical toxicologic evaluation of XR5944 suggests dose-limiting toxicities of reversible myelosuppression and gastrointestinal epithelial damage. Dose escalation follows a modified Fibonnaci search scheme, with 2 patients at each dose level until CTC ≥ grade 2 drug-related toxicity is seen. After the occurrence of such toxicity cohorts of 3-6 patients will be evaluated at that dose level. To establish the dose for Phase 2 studies, 9-15 patients will be treated at the MTD. To date 12 patients have been enrolled, 2 patients each at initial dose levels of 3.6, 7.2, 14 and 24 mg/m$^2$ and one at 36 mg/m$^2$. At the highest level one patient experienced grade III mucositis and one grade IV neutropenia and so 3 patients have been treated at 30 mg/m$^2$ so far without any dose limiting toxicity. The pharmacokinetics of XR5944 are linear over the dose range of 3.6 to 24 mg/m$^2$, with mean (SD) CL = 29.6 (6.4) L/hour/m$^2$, Vss = 937 (382) L/m$^2$, and $t_{1/2}$ = 61.8 (13.4) hours. The average $AUC_{0-t}$ and $C_{max}$ at a dose of 24 mg/m$^2$ are 1060 ng-hr/mL and 1090 ng/mL, respectively. Patient entry continues and updated results will be reported.

### 1.5

### PRECLINICAL EVALUATION OF AW464 (NSC 706704), A NOVEL THIOREDOXIN INHIBITOR

A Mukherjee [1], TD Bradshaw [1], AD Westwell [1], MFG Stevens [1], J Carmichael [2], SG Martin [1]
[1] University of Nottingham, Nottingham, United Kingdom, [2] Astra Zeneca, Cheshire, United Kingdom

AW464 (NSC 706704) is a novel benzothiazole substituted quinol compound active in colon, renal and certain breast cancer cell lines. NCI COMPARE analysis indicates thioredoxin/ thioredoxin reductase signalling as a possible target. Thioredoxin has been shown to be upregulated under hypoxia and through its activity on HIF1α also induces VEGF. This study investigated the potential anti-angiogenic effects, both direct and indirect (*via* altered growth factor production), and altered hypoxic cytotoxicity of AW464. *In vitro* experiments (growth curves, MTS, clonogenic survival assays, ELISA's and RT-PCR) were performed on HT29, SW480 and SW620 colorectal and MCF7 breast cancer cell lines under normoxic and hypoxic conditions. Results were compared against those obtained with normal cell lines, viz. MRCV foetal lung fibroblasts, normal human dermal fibroblasts (NHDF) and neonatal human epidermal keratinocytes (NHEK). Anti-angiogenic effects were studied using large, human umbilical vein and microvessel, human mammary microvessel endothelial cells (HUVEC and HUMMEC, respectively). Results indicate that AW464 exerted anti-proliferative effects on tumour cell lines and endothelial cells with an $IC_{50}$ of ~0.5μM. Of the normal cell lines, only NHDF and MRCV fibroblasts were resistant to the $IC_{50}$ dose. Proliferating, but not quiescent endothelial cells were sensitive to the drug, indicating anti-angiogenic activity. Hypoxia (1% $O_2$ for 48 hours) decreased proliferation and clonogenic survival of colorectal cells at lower drug concentrations ($<IC_{50}$). At these doses, under hypoxic conditions, there was a greater inhibition of VEGF production in HT29 cells. In contrast, bFGF levels were unaffected, suggesting possible involvement of HIF1α. AW464 may be a promising chemotherapeutic drug for colon cancer that may possess enhanced hypoxic potency and also direct and indirect anti-angiogenic activity.

### 1.6

### IMPROVING BREAST CANCER TREATMENT USING NOVEL POLY(ADP-RIBOSE) POLYMERASE-1 (PARP-1) INHIBITORS

SJ Veuger , BW Durkacz
United States, Northern Institute for Cancer Research, Newcastle upon Tyne

NFkB is a stress-inducible transcription complex that induces genes which control proliferation responses and suppress apoptotic cascades. Constitutive activation of NFkB is common in malignant tumours, including breast. PARP-1 is activated as an immediate cellular response to DNA damage. Recent reports have shown reduced NFkB-dependent gene activation in PARP-1 -/- cells suggesting a role for PARP-1 as a co-activator of NFkB.
The effect of IR ± a potent PARP-1 inhibitor (AG14361) on NFkB activity was assessed in breast cancer cell lines which express constitutively activated (MDA-MB-231) or inducible (T47D) NFkB. Once activated, NFkB translocates from the cytosol to the nucleus where it binds to specific NFkB response elements. Following 20 Gy IR, translocation to the nucleus was seen 1h post-IR in the T47D cells (Western blotting) and this was unaffected by 0.4 μM AG14361. Moreover, translocation was also observed in PARP-1 -/- mouse embryonic fibroblasts (MEFs).
The effect of IR ± AG14361 on binding to a NFkB consensus sequence was assessed using an EMSA-based assay. Maximal activation of NFkB by 20 Gy IR was seen after 2h in the MDA-MB-231 cell line. Exposure to AG14361 for 3 h had no effect on constitutive NFkB activity. However, pre-incubation with 0.1 μM AG14361 for 1 h followed by 2h post-IR (20 Gy) incubation at 37°C completely inhibited the IR activation of NFkB in this cell line.
The ability of AG14361 to potentiate IR in these cell lines was investigated. AG14361 sensitised exponentially growing MDA-MB-231 and T47D cells to IR 1.3 and 1.2 -fold**,** respectively**.**
These studies demonstrate an involvement of PARP-1 in the activation of NFkB independent of the signalling cascades through which NFkB is induced and suggest that strategies utilising PARP-1 inhibitors to block NFkB activity in breast tumours should complement existing cancer therapies

### 1.7

### THE ROLE OF P53 IN CHEMOTHERAPY-INDUCED AND DEATH RECEPTOR-MEDIATED APOPTOSIS IN COLON CANCER

U McDermott , L Galligan , DB Longley , PG Johnston
Queen's University Belfast, Belfast, United Kingdom

The tumour necrosis factor family of death receptors are expressed on a variety of human cancers and include Fas/CD95 and the TRAIL receptors DR4 and DR5. Upon binding of their cognate ligands (FasL and TRAIL) these death receptors induce apoptosis, representing a potential target for novel therapeutic agents. A variety of chemotherapeutic agents have been shown to cause up-regulation of the Fas receptor in cancer cell lines. Furthermore, while DR4/DR5 are expressed in both normal and tumour cells, rTRAIL selectively induces apoptosis in tumours. There is increasing interest in the therapeutic potential of agonistic anti-Fas antibodies and recombinant TRAIL (rTRAIL) and how they might increase the cytotoxicity of chemo-therapeutic agents. We examined the therapeutic potential of targeting the Fas and TRAIL pathways in combination with therapeutic agents used in the treatment of colorectal cancer. We have demonstrated by MTT cell viability assay, flow cytometry analysis and PARP cleavage assay a highly significant synergy between the agonistic anti-Fas antibody CH-11 or rTRAIL in the p53 wild-type HCT116 colon cancer cell line co-treated with the chemotherapeutic agents 5-FU, CPT-11 and oxaliplatin. We also observed activation of procaspase 8 when CH-11 or rTRAIL were added after treatment with each of these drugs. In the case of CH-11 treatment, the increased cytotoxicity is associated with induction of the Fas/CD95 receptor. We used the isogenic p53 null HCT116 colon cancer cell line to determine whether these synergistic interactions were p53-dependent. No synergy was observed between CH-11 and 5-FU or oxaliplatin in the p53 null cells. However, significant synergy was observed between CPT-11 and CH-11 in these cells. Interestingly, this did not correlate with Fas induction in response to CPT-11 in the p53 null cell line. Initial results suggest that the synergy between rTRAIL and each of the cytotoxic drugs may not be p53-dependent. These results begin to characterise the role of p53 in regulating death receptor-mediated apoptosis in colon cancer cells following treatment with chemotherapy.

### 1.8

### CELECOXIB TREATMENT LEADS TO DECREASED CYCLOOXYGENASE-2 (COX-2) EXPRESSION IN A XENOGRAFT MODEL OF BREAST CANCER

NLP Barnes [1], F Warnberg [1], D White [2], E Anderson [2], NJ Bundred [1]
[1] South Manchester University Hospital and The Christie Hospital NHS Trust, Manchester, United Kingdom, [2] The Christie Hospital NHS Trust, Manchester, United Kingdom

COX-2 is an inducible enzyme active in the pathway of conversion from arachidonic acid to prostaglandins. Its over-expression is associated with increased early recurrence and decreased survival in breast cancer. COX-2 inhibition has been associated with increased apoptosis and anti-angiogenesis, but no clear surrogate marker has been identified. Our aim was to assess the effect of COX-2 inhibition on tumour growth and COX-2 protein expression in a xenograft model.
Either MCF7/HER-18 or MDAMB231 breast cancer cells were injected into the flanks of nude mice and allowed to form tumours. The mice were then randomised to receive either 0.15% Celecoxib (a COX-2 inhibitor) or control chow. Tumour volumes were measured, and when they reached $1cm^3$ the experiment was ended and tumours were harvested. COX-2 expression was determined immunohistochemically on formalin-fixed, paraffin-embedded tumour sections.

| Cell line | | Median control Growth ($mm^3$) [IQR] | Median COX-2 positive cells (%) [IQR] |
|---|---|---|---|
| MCF7/ HER-18 | Control (n=70) | 100 [35-178] | 65 [60-77] |
| | Treated (n=62) | 39 [4-156]* | 23 [1-60]** |
| MDAMB231 | Control (n=19) | 96 [51-284] | 27 [12-32] |
| | Treated (n=21) | 30 [0-53]*** | 9 [4-18]**** |

[IQR=Interquartile range] *p=0.029 **p=0.0001 ***p=0.0002 ****p=0.046 (Mann Whitney U-Test)
COX-2 is highly expressed in MCF7/HER-18 cells and was seen to be contained within vesicles in MDAMB231 cells.
Administration of Celecoxib significantly decreased both tumour growth and COX-2 protein expression. Therefore, COX-2 expression itself could potentially be used as a surrogate marker of the effects of COX-2 inhibitors.

© 2004 Cancer Research UK 

